# Multichannel near-infrared spectroscopy brain imaging system for small animals in mobile conditions

**DOI:** 10.1117/1.NPh.8.2.025013

**Published:** 2021-06-24

**Authors:** Seung-Ho Paik, Seung Hyun Lee, Ju-Hee Kim, Shin-Young Kang, Zephaniah Phillips V, Youngwoon Choi, Beop-Min Kim

**Affiliations:** a Korea University, College of Health Science, Global Health Technology Research Center, Seoul, Republic of Korea; b KLIEN Inc., Seoul, Republic of Korea; c Korea University, Department of Bio-Convergence Engineering, Seoul, Republic of Korea; d Korea University, Interdisciplinary Program in Precision Public Health, Seoul, Republic of Korea

**Keywords:** near-infrared spectroscopy, small animals, stimulation kit, freely moving, neuroimaging

## Abstract

**Significance:** We propose a customized animal-specific head cap and an near-infrared spectroscopy (NIRS) system to obtain NIRS signals in mobile small animals. NIRS studies in mobile small animals provide a feasible solution for comprehensive brain function studies.

**Aim:** We aim to develop and validate a multichannel NIRS system capable of performing functional brain imaging along with a closed-box stimulation kit for small animals in mobile conditions.

**Approach:** The customized NIRS system uses light-weight long optical fibers, along with a customized light-weight head cap to securely attach the optical fibers to the mouse. A customized stimulation box was designed to perform various stimuli in a controlled environment. The system performance was tested in a visual stimulation task on eight anesthetized mice and eight freely moving mice.

**Results:** Following the visual stimulation task, we observed a significant stimulation-related oxyhemoglobin (HbO) increase in the visual cortex of freely moving mice during the task. In contrast, HbO concentration did not change significantly in the visual cortex of anesthetized mice.

**Conclusions:** We demonstrate the feasibility of a wearable, multichannel NIRS system for small animals in a less confined experimental design.

## 1 Introduction

Small animal models have made significant contributions to enhancing our understanding of many different types of neurological disorders, including Alzheimer’s disease, stroke, and Parkinson’s disease.^1^^–^^4^ Such models allow for experiments in which various parameters can be tightly controlled, from the type of disease to the stimulus provided to the animal. Brain activity in small animals can be measured both non-invasively, such as with positron emission tomography (PET)^5^ or functional magnetic resonance imaging (fMRI),^6^^,^^7^ and invasively, such as with optical microscopy imaging.^8^^,^^9^ However, the use of these modalities for small animal models is limited because they typically require immobilization during imaging.

Near-infrared spectroscopy (NIRS) is a proven neuroimaging technique for non-invasive and real-time monitoring of cerebral oxygenation changes.^10^ It offers advantages over other modalities such as cost-effectiveness compared with PET and fMRI and greater robustness for motion artifacts than electroencephalogram.^11^ Most importantly, NIRS can be constructed in the form of wearable probes that do not obstruct natural movements.^12^^,^^13^ Due to these advantages, wearable NIRS probes have already been developed and used for animals.^14^^–^^22^ However, animals are still stressed by fixation, which may subsequently impact brain function,^23^^,^^24^ whereas the use of anesthesia restricts specific behavioral and cognitive experiments since anesthetics strongly modulate the functional properties of cortical neurons.^25^^–^^28^ Further, these studies mainly focus on one or two candidate brain regions and are biased toward regions previously shown or predicted to be involved in a given behavior. An unbiased brain-wide map during animal behavior could lead to a system-level understanding of how brain activities are affected by behavior and could guide the selection of the brain regions to be studied. However, current NIRS studies have limited resolution for revealing activity in small brains and are difficult to apply to awake and behaving small animals. Although most studies employ 400  μm fiber-optic cables, it is difficult to measure multichannel signals in small brains because the source–detector distances must be maintained at around $50\;{\mathrm{mm}}^{2}$. In addition, it is difficult to fix the fibers on the brain, so measurements are obtained head-fixed. Therefore, a new type of NIRS implementation that not only is non-constraining for an awake animal and robust to potential motion artifacts but also allows for real-time functional imaging is required.

To address the abovementioned challenge, we developed and tested a long-fiber-based, multichannel continuous-wave (CW)-NIRS system that uses a customized light-weight head cap attached to the mouse’s skull. The experiment was performed in a closed-box stimulation kit for unrestrained animals in which animals are allowed to move around freely in the box. The performance of the developed CW-NIRS system to monitor oxygenation changes in the cortex was validated by visual stimulation in anesthetized (N=8) and freely moving mice (N=8). Our aim was to demonstrate the ability of our NIRS system to monitor cortical hemodynamic changes in small animals in both anesthetized and freely moving states.

## 2 Materials and Methods

### 2.1 Instrumentation

**Fig. 1:**
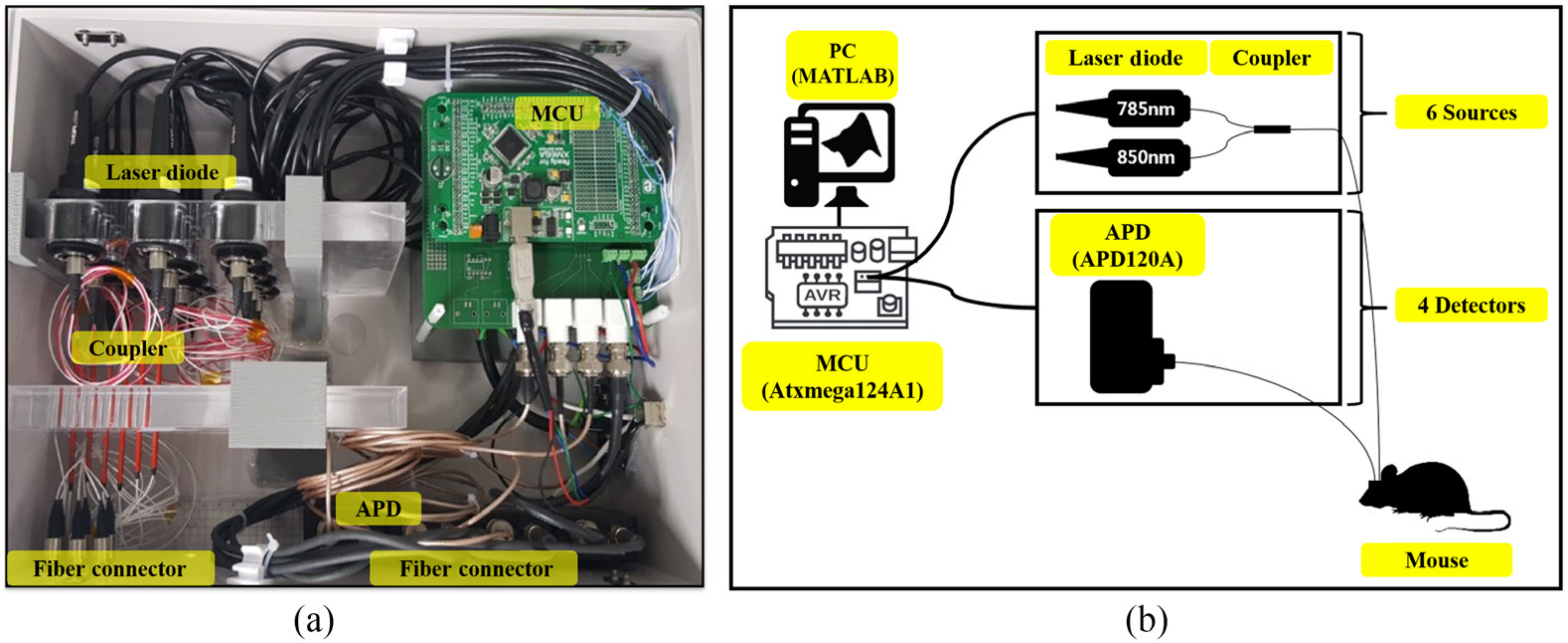
Custom-made CW-NIRS system and experimental setup: (a) customized CW-NIRS system consisting of an MCU, laser diode, coupler, APD, and optical fiber connector and (b) schematic diagram of the experiment. CW-NIRS, continuous-wave near-infrared spectroscopy; MCU, microcontroller unit; APD, avalanche photodiodes; and PC, personal computer.

The customized CW-NIRS system [Fig. 1(a)] was composed of illumination and detection components to acquire transient light intensity changes from the mouse. The sources of the CW-NIRS systems consisted of six pairs of dual-wavelength (785 and 850 nm) laser diodes with collimation tubes and optics (LT240P-B, Thorlabs). Each pair of collimated lights was combined into a long and light-weight glass optical fiber (105  μm in core diameters) with a multimode coupler to reduce the number of optical fibers. For greater sensitivity, four avalanche photodiodes (APDs) (APD120A, Thorlabs), also connected to optical fibers, were used with a customized amplifier and low-pass filter circuit to remove high-frequency noise. Each source switching and detecting sequence was controlled by a 16-bit microcontroller unit (MCU) (Atxmega128A1, AVR). The sampling rate was 5 Hz and the system communicated via serial communication through MATLAB (MathWorks, Inc., Natick, MA, USA). Figure 1(b) shows the overall schematic diagram of our implementation.

### 2.2 Animal Preparation

In total, 16 male C57BL/6 mice (age 9 weeks, Orient Bio Inc., Seongnam, Republic of Korea), weighing 25 to 27 g, were used in this experiment. This experiment was approved by the Korea University Institutional Animal Care and Use Committee (IACUC-2018-49) and carried out in accordance with the Korea University guidelines. Before any surgical procedure, 2% to 5% isoflurane was administered to the mouse until it was anesthetized; then the mouse was placed in a stereotaxic frame. To maintain the mouse’s body temperature, a heating pad (maintained at 37°C±1°C) was placed under the body, and the head was tightly fixed using a nose cone and ear bars. Stereotaxic coordinates were determined with respect to the bregma. The scalp was first shaved, disinfected with ethanol, and then removed with spring scissors. The flaps were removed to make the skull visible. The cranial surface was carefully cleaned with 4% alcohol and scraped to expose ∼1.5×1.0  cm of the skull. The duration of the surgical procedure was ∼15  min. Thereafter, the customized 3D head cap printing process was immediately started.

### 2.3 3D-Printed, Customized Head Cap

**Fig. 2:**
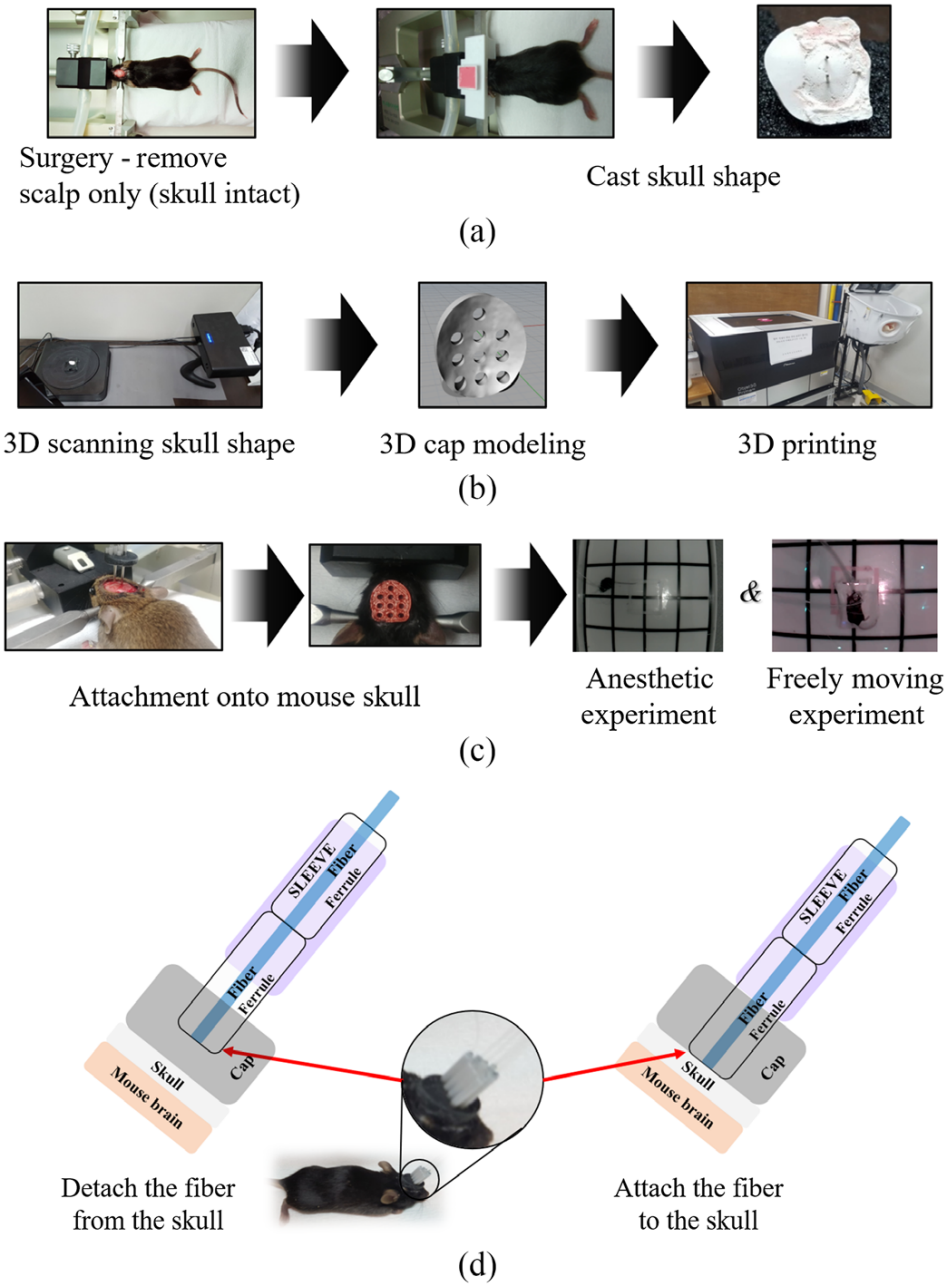
Customized 3D head cap printing technique: (a) a cast of the skull was formed using alginate and plaster; (b) the mold was converted to a 3D mesh; (c) the 3D-printed head cap with customized channel layout was placed on the mouse skull (Video 1, MP4, 8.4 MB [URL: https://doi.org/10.1117/1.NPh.8.2.025013.1]; Video 2, MP4, 5.7 MB [URL: https://doi.org/10.1117/1.NPh.8.2.025013.2]); and (d) a ferrule-sleeve system was used for repeated experiments.

To ensure stable attachment of the optical fibers onto the mouse skull, we developed a novel 3D printing technique to accommodate the skull’s size and shape. After surgery, to prepare a 3D customized head cap, the shape of the skull’s surface was acquired using a customized molding frame made of alginate and plaster [Fig. 2(a)]. The molded object was scanned using a 3D scanner (EinScan Pro, Hustem Co., Ltd., Gangnam-gu, Seoul) to acquire the 3D mesh. The mesh was refined using a 3D design program (Rhino 3D, Rhinoceros, Robert McNeel & Associates, Seattle, WA), including placement of the probe holes (distanced ∼2.5  mm apart) [Fig. 2(b)], and then it was 3D printed. For customized 3D head cap printing, we used a stereolithography apparatus 3D printer using a biocompatible ultraviolet curable polymer (3DMaterials). The head cap was bonded to the skull using biocompatible dental glue (Super Bond C&B, Sun Medical Co., Ltd., Shiga, Japan) [Fig. 2(c)]. After hardening, optical fibers were inserted into the holders and fixed. Ferrules and sleeves were used to prevent twisting and minimize damage to the optical fiber by biting or grooming. By separating ferrule and sleeve, the animals could perform repeated experiments [Fig. 2(d)]. The total weight of the printed head cap and the long optical fibers was ∼1  g.

**Fig. 3:**
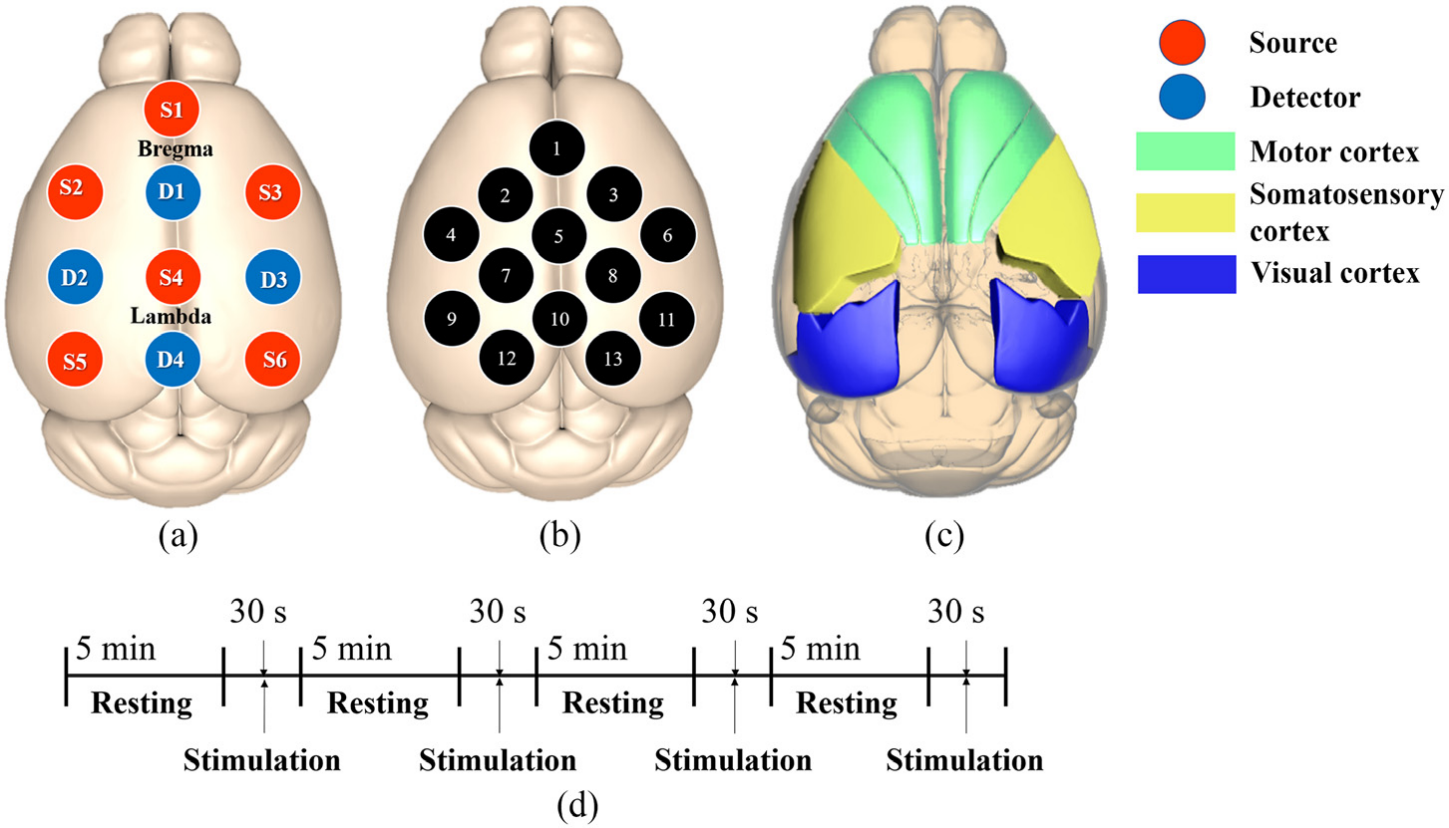
Customized head cap and estimated channel position on a model brain and experimental protocol: (a) estimated probe position overlayed onto a 2D model of the mouse’s brain; (b) estimated channel position according to the source-detector layout; (c) regions of the mouse cortex; and (d) experimental protocol.

Since the 3D rendering software determines probe position, the optical fiber layout can easily be modified depending on the experiment’s purpose. This study fixed the optical fibers [six sources and four detectors, Fig. 3(a)] to target the visual and somatosensory cortex in the mouse brain [Fig. 3(b)]. This layout resulted in a total of 13 NIRS channels [Fig. 3(c)].

Preparing a 3D head cap took approximately an hour for each mouse. It took about an hour and a half from surgery until head cap attachment. Thereafter, the anesthesia group immediately started the experiment, but the freely moving group started the experiment the next day after awakening from the anesthesia, adaptation, and stabilization.

### 2.4 Visual Stimuli

A visual stimulation task was chosen to validate our system by monitoring the mice’s hemodynamic response. A stimulation kit was designed to verify the customized CW-NIRS system. The stimulation kit consisted of a rectangular-shaped box (590  mm×590  mm×610  mm) made of black acrylic. In total, 16 mice were divided into two arbitrary groups for the task, including the freely moving group (N=8) and anesthesia group (N=8). The experiments were arranged in a block paradigm, consisting of four rest periods (300 s) and four stimulation periods (30 s) [Fig. 3(d)]. On the inside of each wall, green LEDs (490 to 510 nm, LXZ1-PE01, Lumileds LLC, San Jose, CA) were attached to use as light sources for the visual stimulus. The optical fibers reach into the stimulation kit through a hole (diameter 80 mm) in the upper side of the box, allowing them to move following the mouse. The fiber length was 1 m, long enough to allow a mouse to freely move within the stimulation kit area (590  mm×590  mm), which is sufficient for visual stimuli tasks.

In the anesthesia group, each mouse was anesthetized using 2% to 5% isoflurane and 100% $O_{2}$ inhaled through anesthesia tubes during the task, and they were placed in the middle of a stimulation kit. Experiments typically lasted 1 h and were followed by euthanasia. For experiments in awake mice, we placed the mouse on the middle of the customized stimulation kit, and experiments started immediately after placing them on the box. The mice were allowed to move freely in the stimulation kit while the green LEDs presented the visual stimulus. The unanesthetized mouse was allowed to move freely without any barriers in the closed box. All tasks were monitored and recorded by an infrared camera (5-MP Night Vision Camera for Raspberry Pi, SEN0184, DFRobot, Shanghai, China) placed on the top of the kit (Videos 1 and 2).

### 2.5 Data Processing and Analysis

All analyses were performed using custom scripts written in MATLAB. Two time-series light intensity changes were acquired at each wavelength to calculate oxyhemoglobin (HbO) and deoxyhemoglobin (HbR) concentration changes from optical density according to the modified Beer–Lambert law.^29^ The hemodynamic changes were time-filtered using a 0.009 to 0.08 Hz first-order Butterworth band-pass filter to remove high-frequency noise that could affect the analysis while also maintaining important spontaneous hemodynamic fluctuations.^30^^–^^32^ Brain activation map images were spatially smoothed with a Gaussian filter using a sigma of 1.25 mm. The grand block average of HbO and HbR concentration changes was obtained by averaging over all mice with a stimulation-related response in each group. To compare cortex activity, we used the mean values of the concentration changes in HbO and HbR (meand ± SD) obtained from all channels in each group. The mean HbO and HbR values were compared within channels using a paired t-test to determine the differences in HbO and HbR changes between corresponding channels. To verify the significance of differences between freely moving and anesthesia groups, the mean HbO and HbR values were compared across groups using a two-sample t-test. The significance level was set at p<0.05.

## 3 Results

### 3.1 Cortical Hemodynamic Responses

**Fig. 4:**
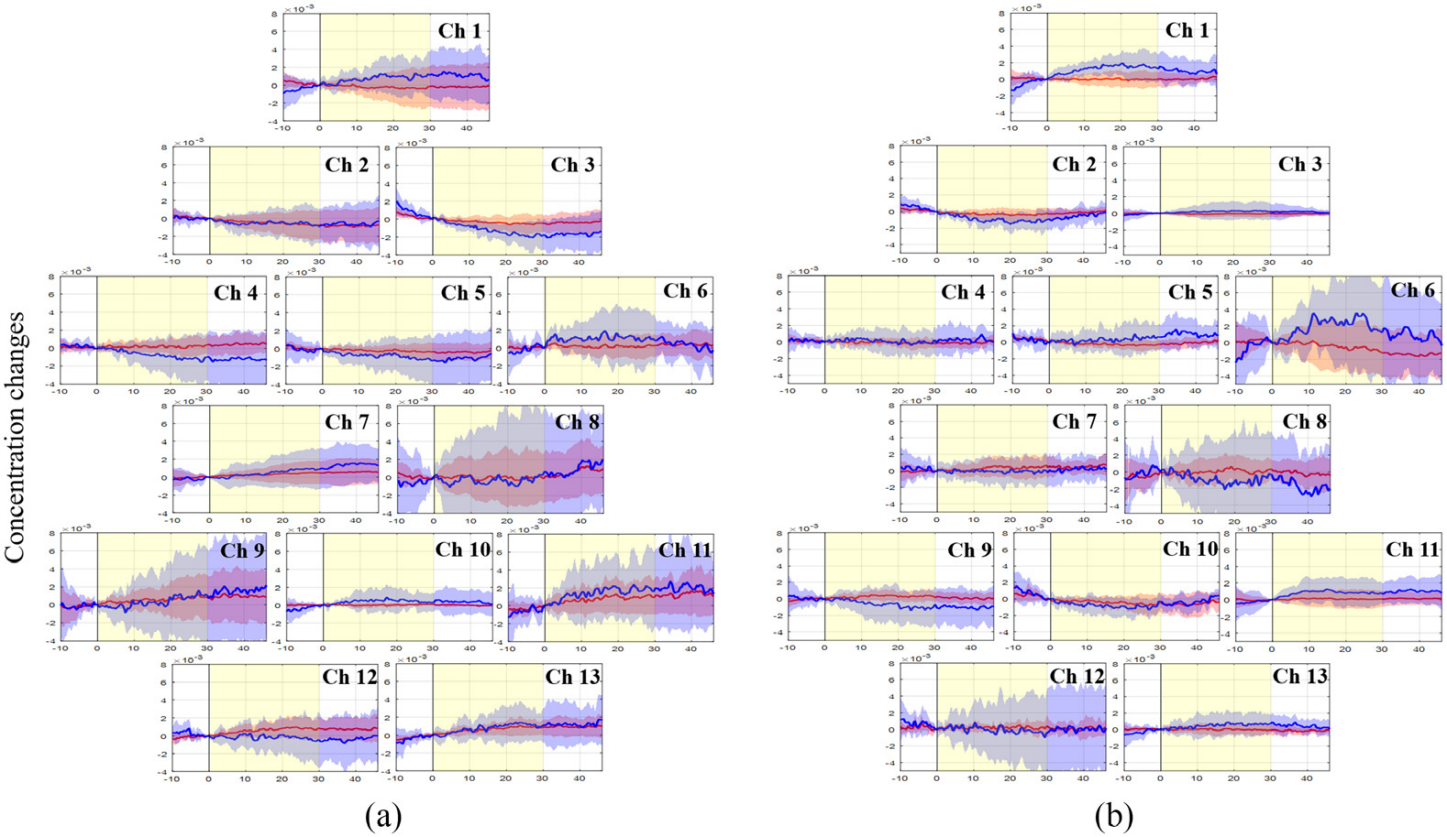
Time course of changes in HbO and HbR concentration during the visual stimulation task in each channel. The horizontal axis represents time course. The vertical axis represents changes in HbO and HbR concentration. The solid line (HbO: red; HbR: blue) shows hemodynamic responses. The black solid line shows the stimulation start time. Yellow box indicates the stimulation period. Differences in HbO response between mice in a freely moving state or under anesthesia during visual stimulation: (a) freely moving group and (b) anesthesia group. HbO, oxyhemoglobin; HbR, deoxyhemoglobin; and CH, channel.

The grand average waveforms of HbO and HbR concentration changes during the visual stimulation task in both groups are shown in Fig. 4. The grand average of the HbO in the freely moving group slightly increased in the visual cortex (channels 9, 11,12, and 13) during the task period, whereas that in the anesthesia group did not show substantial changes. In the freely moving group, the HbO responses for movements could not be observed in the motor cortex (channels 1, 2, 3, and 5) because the hemodynamic responses following movement were filtered to clearly identify those in the visual cortex (see the Supplementary Material). In both groups, HbR signals had a higher hemodynamic variability during the task, which corresponds to random noise but would not removed by filtering.

### 3.2 Imaging of Hemodynamic Changes

**Fig. 5:**
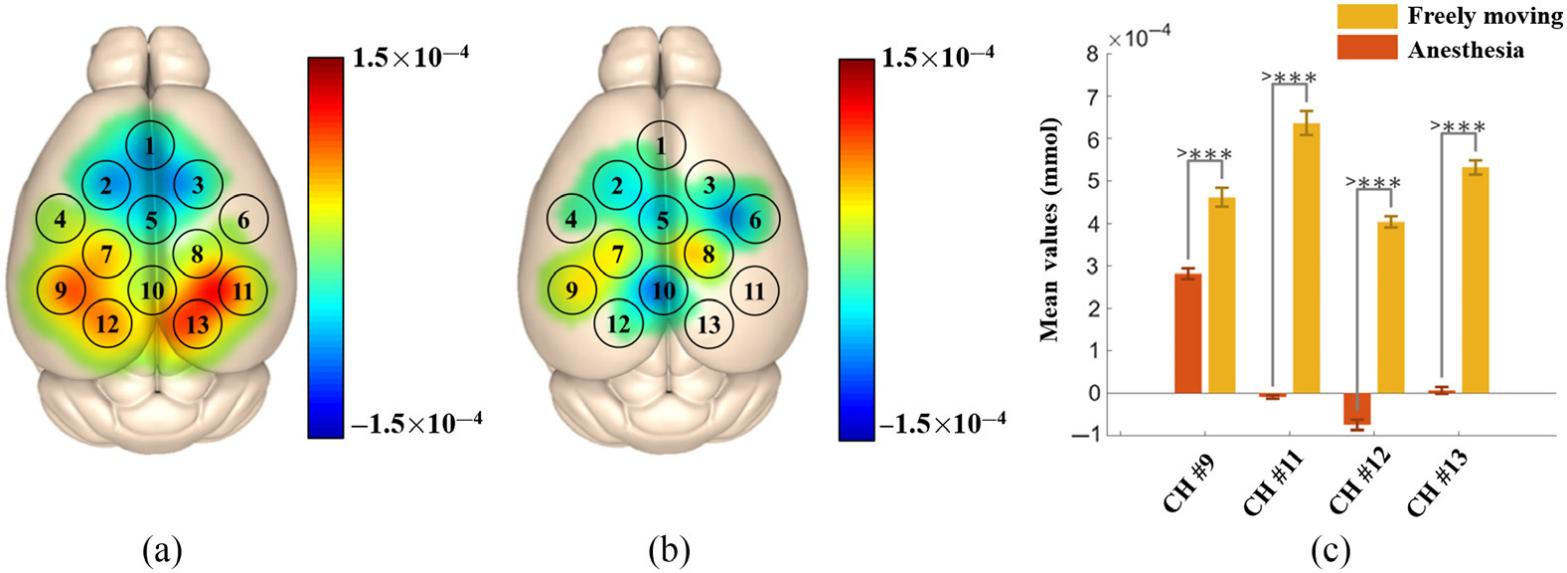
Differences in HbO response between mice in a freely moving state or under anesthesia during visual stimulation: (a) 2D HbO image of freely moving mice during visual stimulation; (b) 2D HbO image of anesthetized mice during visual stimulation; and (c) statistical results of mice in a freely moving state or under anesthesia during visual stimulation. HbO, oxyhemoglobin and CH, channel.

Figure 5 shows cortical activation maps and group averaged HbO for the visual stimulation task in freely moving and anesthetized mice. The HbO response increased in the visual cortex (channels 9, 11, 12, and 13) when visual stimulation was performed for freely moving animals [Fig. 5(a)]. Under anesthesia, the HbO response increased in channels 9; however, there were no significant changes in the HbO response for channels 11, 12, and 13 [Fig. 5(b)]. The mean values of HbO concentration changes were compared between the freely moving state and anesthesia [Fig. 5(c)]. Visual stimulation tasks induced significantly higher activation in the visual cortex (channels 9, 11, 12, and 13) in the freely moving group than in the anesthesia group. Table 1 shows the mean HbO values and statistical difference between the freely moving and anesthesia groups. All channels in the visual cortex had a significant difference for HbO (p<0.001). The p-value confirmed the visual analysis of results reported in Figs. 5(a) and 5(b). The averaged hemodynamic responses in channels 11, 12, and 13 in the anesthesia group had a very small amplitude as compared with those in the freely moving group, indicating no visual stimulation was received under anesthesia.

**Table 1:** Average activation in brain regions during visual tasks in HbO.

Channels	Groups		t-value	p-value
	Freely moving	Anesthesia		
9	$4.616\times 10^{-4}\pm 1.24\times 10^{-5}$	$2.81\times 10^{-4}\pm 2.20\times 10^{-5}$	1.97	$1.64\times 10^{-11}$*
11	$6.362\times 10^{-4}\pm 3.93\times 10^{-6}$	$-9.38\times 10^{-6}\pm 2.82\times 10^{-5}$	1.97	$1.04\times 10^{-48}$*
12	$4.036\times 10^{-4}\pm 1.19\times 10^{-5}$	$-7.46\times 10^{-5}\pm 1.28\times 10^{-5}$	1.96	$2.77\times 10^{-79}$*
13	$5.318\times 10^{-4}\pm 8.31\times 10^{-6}$	$6.65\times 10^{-6}\pm 1.64\times 10^{-5}$	1.97	$2.15\times 10^{-72}$*

Note: Values are mean ± standard error.

* p<0.001.

## 4 Discussion and Conclusion

In this study, we developed a CW-NIRS system and stimulation kit for functional brain monitoring of freely moving small animals in a constrained paradigm and validated the system through simple stimulus experiments. As a result, we found that visual stimulation induced HbO concentration changes in the visual cortex in freely moving mice compared with anesthetized mice. These changes in HbO have been well described in the literature on fundamental physiological neurovascular coupling following neural activation and validated in animal experiments.^33^^–^^36^ The agreement of our current result of cortical activation with similar studies demonstrates the potential of our system to be used as a monitoring tool for the brain mechanisms of small animals in a freely moving state.

Previous NIRS studies on small animals were generally performed under anesthesia or with their heads restrained to avoid motion artifacts;^37^^–^^39^ however, these conditions can profoundly affect the neurovascular response due to their extensive action on nerve and vascular reactivity and baseline physiology.^40^ These limitations are particularly evident in mice with poor reproducibility and weak neurovascular reactions.^41^^–^^43^ Compared with other studies of functional brain imaging techniques for small animals, our system is easily adaptable to animals in a freely moving state. Our experimental setup of individualized head caps allows the optical fiber to make solid contact with the skull and provides minimal tension as the animal moves. These advantages provide an environment where the animals can move freely in the stimulation kit, making it suitable for functional brain studies in freely moving animals. Our system also removes the scalp, allowing us to observe the intact cortex using a minimally invasive method and allows for repetitive imaging of the cortex of small animals. Additionally, our system can measure multiple cortex areas in non-immobilized animals. Previous animal studies using functional NIRS could only measure in limited areas,^22^^,^^44^ since using 400-μm core diameter optical fiber to multichannel imaging in the brain of small animals was difficult. In this study, the use of 105  μm core diameters optical fibers allowed for multichannel imaging and hemodynamic response measurements in a wide area. Therefore, it can be used as an alternative method to observe hemodynamic changes in small animals. However, it is difficult to make a suitable head cap for the curved surface of the brain because of the spacing between optical fibers that is required to measure blood flow in the cortex (especially those targeting the temporal lobe for auditory tasks). In addition, our chosen fiber length and thickness, 1-m long and only 105-μm core diameter, respectively, creates light loss, and since the mouse’s brain is small, the signal-to-noise ratio (SNR) was not good because light penetration depth can increase at our probes’ position. Therefore, the hemodynamic responses did not clearly appear in the stimulation period, and random noise and hemodynamic variability were high in HbR. Nevertheless, our study demonstrated the potential of NIRS for small animals.

The light-weight head cap is ideal for improving SNR and measuring cerebral blood flow in small animals. However, our CW-NIRS system can still improve. The size and weight of the head cap could be reduced using light-weight 3D printing material and lower thickness head cap designs. Our system was designed to measure brain signals in multiple cortex regions, but we only evaluated visual cortical regions in this study. Therefore, this is a preliminary study for system validation, and future studies using more stimuli and conditions are warranted.

Our study demonstrated that the proposed CW-NIRS system allows for better detection of brain function in freely moving small animals using minimally invasive methods. Although this study was limited due to the small number of mice, we demonstrated the feasibility of monitoring cerebral oxygenation changes in freely moving small animals using CW-NIRS. Further research will confirm whether the CW-NIRS system can be used as a monitoring tool for various animal disease models and provide insight into animal behavior.

## Code, Data, and Materials Availability

The NIRS data used to support the findings of this study are open and available from the corresponding author upon request.
