# Supplementary material for: Multichannel near-infrared spectroscopy brain imaging system for small animals in mobile conditions
Source: Neurophotonics. 2021 Jun 24;8(2):025013. doi: 10.1117/1.NPh.8.2.025013 (PMC8230091; doi:10.1117/1.NPh.8.2.025013)
Supplement: Supplementary file 1 [file NPh_008_025013_SD001.pdf]

Motor activity during visual stimulation

In the visual stimulation experiment, the concentration changes in HbO for mouse movement could not be shown in the motor cortex. The reason is that movement signals were removed to identify the HbO response of the visual area. The following is the result of lowering the filtering range. Noise is mixed a little, but activity appears in the visual cortex and motor cortex when the mouse shows movements.

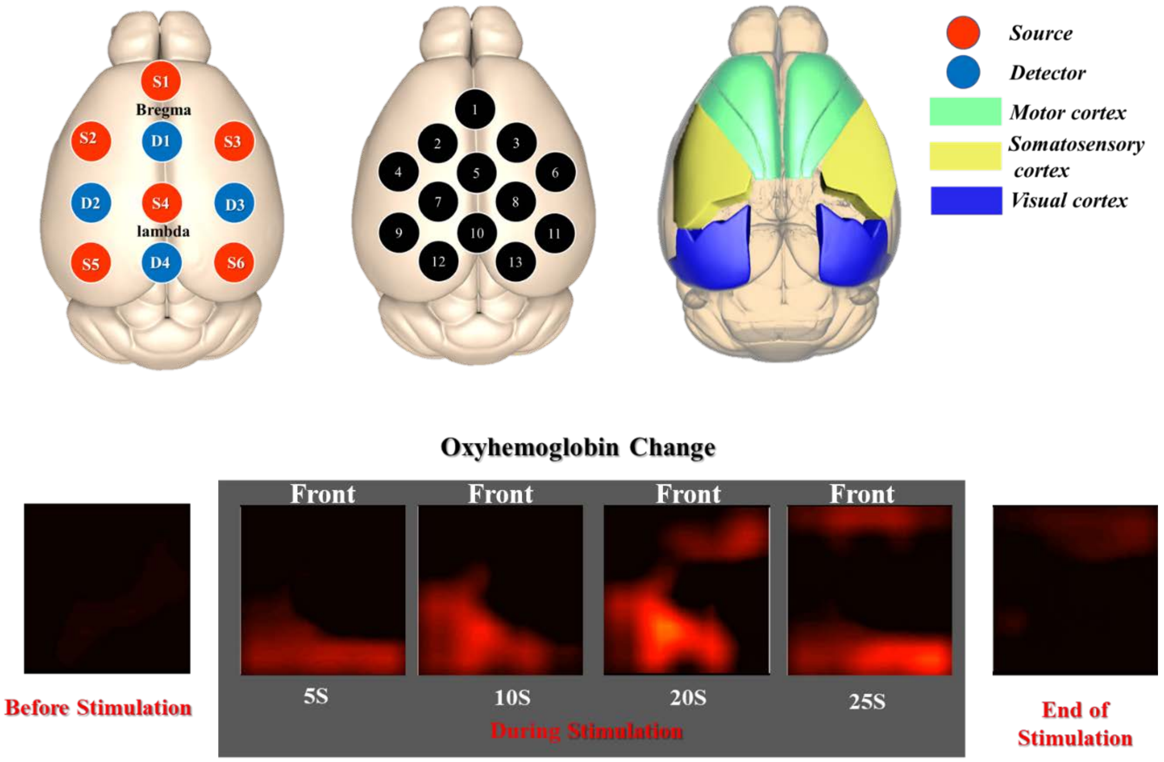

Fig. 1 HbO responses in motor and visual cortex during stimulation.
